# The spatial and temporal components of functional connectivity in fragmented landscapes

**DOI:** 10.1007/s13280-014-0588-6

**Published:** 2015-01-09

**Authors:** Alistair G. Auffret, Jan Plue, Sara A. O. Cousins

**Affiliations:** Landscape Ecology, Department of Physical Geography and Quaternary Geology, Stockholm University, 106 91 Stockholm, Sweden

**Keywords:** Conservation, Dispersal, Global change, Historical ecology, Landscape change, Semi-natural grassland

## Abstract

Connectivity is key for understanding how ecological systems respond to the challenges of land-use change and habitat fragmentation. Structural and functional connectivity are both established concepts in ecology, but the temporal component of connectivity deserves more attention. Whereas functional connectivity is often associated with spatial patterns (spatial functional connectivity), temporal functional connectivity relates to the persistence of organisms in time, in the same place. Both temporal and spatial processes determine biodiversity responses to changes in landscape structure, and it is therefore necessary that all aspects of connectivity are considered together. In this perspective, we use a case study to outline why we believe that both the spatial and temporal components of functional connectivity are important for understanding biodiversity patterns in the present-day landscape, and how they can also help us to make better-informed decisions about conserving and restoring landscapes and improving resilience to future change.

## Introduction

Connectivity in ecology is traditionally defined as how the movement of various ecological units or entities is facilitated by their surroundings. Connectivity is therefore important for understanding and managing ecological systems, and the relationships between individuals, populations, and communities with the surrounding habitats, landscapes, and regions which they inhabit (Taylor et al. 1993; Hanski 1999; Leibold et al. 2004). During the 20th century, human activity has impacted connectivity through land-use change and associated habitat destruction and fragmentation, posing serious threats to biodiversity worldwide (Sala et al. 2000).

As habitat is lost, landscapes become more fragmented and less connected, usually with negative effects on biodiversity (Fahrig 2003). As populations and communities become increasingly isolated, the likelihood of dispersal between them decreases. According to metapopulation theory (Hanski 1999, 2011), a reduction in dispersal can decrease opportunities for locally extinct species to re-colonize, leading to diversity losses at larger scales (Ozinga et al. 2009). In the longer term, reduced connectivity can have reinforcing negative effects on a population’s long-term viability through the erosion of genetic diversity (Lienert 2004). The loss of connectivity on landscape scales (tens of square kilometers) also has consequences at regional and international scales (hundreds of square kilometers and more), as connectivity is an important prerequisite for species to move the long distances required to avoid extinction due to anthropogenic climate change (Krosby et al. 2010).

It is clear that connectivity is a key concept relating to the ecological effects of environmental change. Currently, connectivity science is focused on how habitats and organisms are connected in space (for an in-depth overview of ecological connectivity, see Crooks and Sanjayan 2006). With a focus on habitat fragmentation, the aim of this perspective is to highlight that species can also exhibit temporal responses to environmental change, and thus that connectivity can exist in time as well as space. We argue that an integrative view of connectivity, including both the spatial and temporal aspects of functional connectivity alongside structural connectivity can be useful for understanding ecological responses to environmental change, particularly habitat destruction. We will first broadly define the different types of connectivity before using a case landscape to demonstrate why we believe that our approach can aid the understanding and management of biodiversity following land-use change and habitat destruction.

## Definitions of connectivity

### Structural connectivity

Pure structural connectivity is a general measure of connectivity related to the physical characteristics of the landscape, without any consideration of the attributes of any potential organisms of interest (Tischendorf and Fahrig 2000). This can be measured in several ways (see Calabrese and Fagan 2004), most commonly with regard to the area of suitable habitat within a certain proximity to a focal patch, or the distance between habitat patches (Moilanen and Nieminen 2002). Structural connectivity can also take the form of linear corridors (Haddad et al. 2003) or stepping stones (Baum et al. 2004) between target habitats. Habitat destruction results in a decrease in structural connectivity by reducing habitat area and increasing isolation (Fahrig 2003), and an increase of both the area and connectivity of target habitats through restoration is required to facilitate ecological responses to change, both now and in the future (Hodgson et al. 2009). However, structural connectivity is ultimately based on the human perception of a landscape and may not always be an accurate gauge of how ecological populations and communities use their physical environment (Fischer and Lindenmayer 2007).

### Functional connectivity

Functional connectivity is the response of organisms to the various elements of the landscape (Tischendorf and Fahrig 2000), and is therefore highly dependent on the organisms and landscape being studied. While human activity is usually the cause of changes in structural connectivity through land-use change, it is functional connectivity which determines the ecological effects of habitat destruction and fragmentation.

#### Spatial functional connectivity

Functional connectivity is generally considered in terms of realized movement in space. In mobile species such as butterflies, birds, and larger mammals, areas of the landscape can be functionally connected through the movement of individuals (Schooley and Wiens 2003), while plant populations and communities are considered to be functionally connected when effective seed or pollen dispersal occurs between them (e.g., van Geert et al. 2010; Auffret and Plue 2014). Plants, insects, and mammals have all been observed to move between structurally connected habitat patches (Haddad et al. 2003), and several methods have been developed to more accurately predict functional connectivity by combining structural connectivity and the attributes of study species. Graph-theoretical models of connectivity (Urban and Keitt 2001) and network analysis (Bodin and Norberg 2007) both use habitat configuration and species dispersal ability to measure connectivity in fragmented landscapes, while the movement and behavior of species in relation to their physical environment can be useful in understanding the connectivity of animal populations and the plant species they disperse (Nathan et al. 2008; Perea et al. 2013; Auffret et al. 2014).

#### Temporal functional connectivity

Although movement in space is important, ecological processes relevant to populations and communities involve an interaction of both space and time (Alexander et al. 2012). Where functional connectivity has usually been described as movement in space, persistence in time can allow an individual or population to remain in the same place despite a lack of spatial continuity of habitat or environment, or allow a delayed response following connectivity in space. We believe that such temporal responses are important in determining functional connectivity, and we therefore define the temporal component of functional connectivity as the persistence of organisms in time, in the same place.

Examples of this temporal component include the persistence of plant and animal species following changes in climate and land use (Eriksson 1996; Baker et al. 2008), while propagules are able to remain dormant for a period of time in seed banks or egg banks until suitable living conditions arrive (Thompson and Grime 1979; Mergeay et al. 2007). Temporal responses to environmental change have been observed in a wide range of organisms in various habitats (Kuussaari et al. 2009), whereby species have persisted in a landscape despite losses in habitat structure and quality. Temporal functional connectivity can thus provide a kind of insurance against environmental variability a local scales, but also result in delayed immigration or recolonization of suitable habitats (Jackson and Sax 2010).

The elements of temporal connectivity we describe show that it is not necessarily a new concept, but the presence of remnant populations, communities, and dormant propagules is often undervalued in ecology and conservation. We believe that it is valuable to frame these temporal processes together with the spatial component of functional connectivity to improve understanding of the ecological responses to changes in landscape structure over periods of environmental change. In the following section, we use examples from an agricultural system which has undergone changes in structural connectivity to discuss the importance of considering both the spatial and temporal components of functional connectivity for biodiversity and conservation management in semi-natural grassland plant communities. The long generation times in many typical grassland plants mean that the temporal aspect of connectivity is often especially relevant, while plants provide the habitat structure important for the entire suite of terrestrial organisms in the landscape with which they interact.

## Connectivity in practice: A case landscape

### Semi-natural grasslands

Semi-natural grasslands are grassland habitats which have been the subject of low-intensity management as pasture or meadow under time periods of several hundreds to thousands of years, without the substantial addition of artificial fertilizers or herbicides (Pedersen and Widgren 2011). The high species richness of European grasslands is thought to be the result of the gradual accumulation of plant species due to the historically large areas of habitat with a long management continuity (Eriksson 2013). Unfortunately, agricultural intensification has reduced the number and area of these grasslands, and those that remain are still under threat (WallisDeVries et al. 2002).

In this section, we will use semi-natural grasslands as a basis to illustrate our rationale regarding how an integrative approach on connectivity may improve our understanding of how various drivers of land-use change have shaped patterns of species diversity in fragmented landscapes. We use an example landscape of a 25 km^2^ area on the western side of the island of Selaön in southeastern Sweden (59°24′ N, 17°10′ E), where structural, and the spatial and temporal components of functional connectivity in semi-natural grassland fragments have been investigated separately (e.g., Cousins and Eriksson 2008; Plue and Cousins 2013; Auffret and Plue 2014). Supplementing such studies with examples from our other work and the wider literature, we aim to collectively assess and understand the threats of, and community responses to changes in connectivity, and how they can be managed to maintain and enhance ecological resilience to environmental change in rural landscapes.

### Structural connectivity

**Fig. 1:**
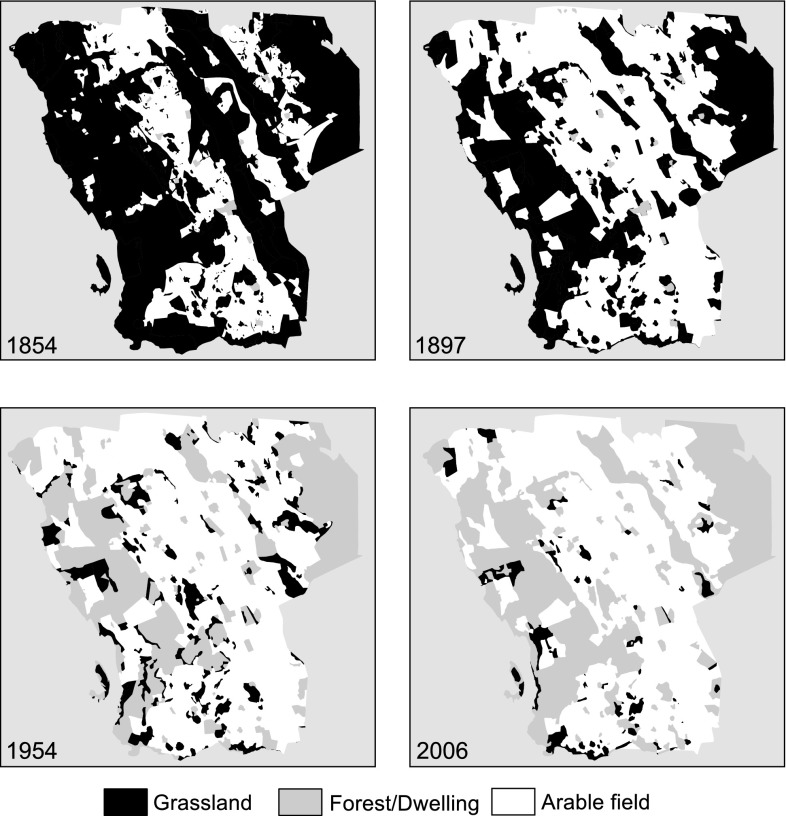
Changes in broad land-use categories over four time-steps in an area of Selaön, southeastern Sweden. Land cover digitized and interpreted from old cadastral maps in 1854 and 1897 and from aerial photographs in 1954 and 2006

In rural landscapes, humans, more specifically farmers and policy-makers, are the major drivers of structural connectivity by means of their decisions on landscape management (Kininmonth et al. 2015). Therefore, the total area of semi-natural grassland habitat, the size distribution of remnant fragments, and their physical configuration can provide an example of how humans shape the physical landscape and consequently structural habitat connectivity. Like much of agricultural Sweden, Selaön is an old cultural landscape, with continuous and relatively static human occupation for around 2000 years. Available records show that management remained quite stable until the mid-late 19th century and the beginning of the agricultural revolution (Dahlström et al. 2006). Forest grazing outside village boundaries was gradually replaced by permanent fodder production and grazing on arable fields. Mown grasslands on deep moist soils were drained and converted to arable fields, whereas grasslands on poorer soils later became afforested (Fig. 1; Dahlström et al. 2006; Cousins and Eriksson 2008). These changes are representative of the changes occurring in the surrounding region (Cousins et al. 2015).

**Fig. 2:**
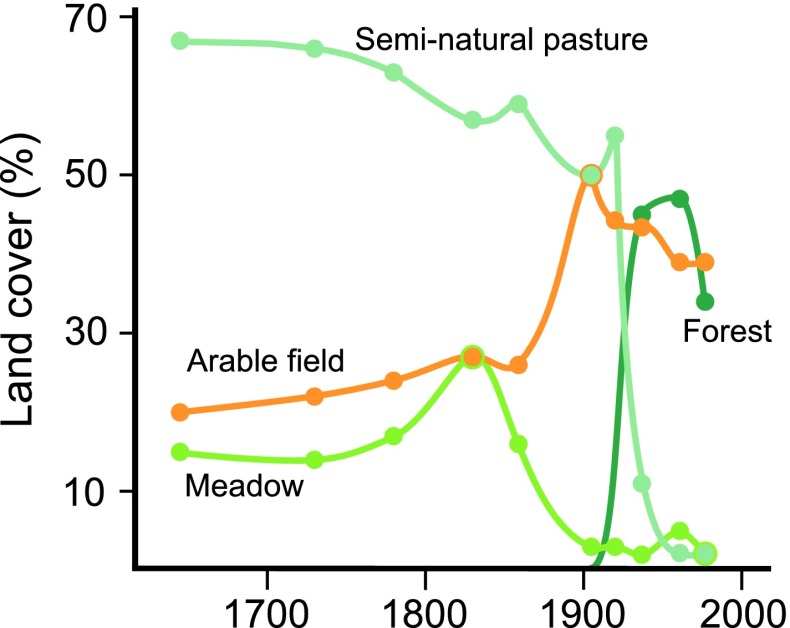
Relative changes in land-use categories on the island of Selaön (95 km^2^), southeastern Sweden 1626–1972. Plotted using data from Dahlström et al. (2006)

**Fig. 3:**
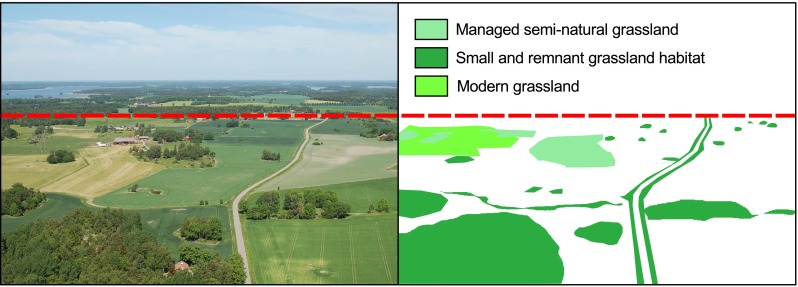
Present-day photograph from an area of Selaön, southeastern Sweden (*left*) with interpretation of grassland habitat (*right*, until *dashed line*). Very little managed semi-natural grassland is left, but grassland communities can still persist in both abandoned grasslands of various sizes and in road verges. Modern grasslands are less species-rich than historical grasslands, but all additional features can contribute to increased connectivity in the landscape

These land-use trajectories naturally have diverse effects on grassland structural connectivity. Most pervasive is the large decline in grassland area due to the direct conversion to arable fields and forest plantations, and the more indirect forest succession resulting from grazing abandonment (Figs. 1, 2). This habitat loss is magnified as remaining grasslands are smaller, interpatch distances larger, and the intensive agri- and silvicultural management results in a more hostile matrix. Plant species richness declines strongly following grassland abandonment (Cousins and Eriksson 2008), while conversion to agri- and silviculture effectively eliminates grassland communities. What is left besides a limited number of smaller grasslands are small bedrock outcrops and linear habitats which can act as small refugia for a subset of robust and drought-tolerant grassland species in an otherwise hostile landscape (Fig. 3; Cousins 2006). Although marginal in surface area, they act as reservoirs of species diversity, an effect most pronounced in highly fragmented landscapes such as Selaön (Lindborg et al. 2014), while linear elements can additionally provide structural connectivity between grassland habitats (Auffret and Cousins 2013). As losses in structural connectivity often provide the background to the functional responses of organisms, this dramatic change in the availability of semi-natural grassland will have significant effects on the long-term survival of plant species reliant upon these grassland habitats.

### Functional connectivity

#### Spatial functional connectivity

For the vast majority of terrestrial plant species, spatial functional connectivity entails movement between suitable habitat patches during the seed stage of its life-cycle. In fragmented landscapes, mobility—or the capability of a plant to disperse their seeds between suitable habitats—is key to the maintenance of vital (meta) populations, and thus to long-term survival. Despite seed dispersal being a crucial process even in non-fragmented habitats (Vandvik and Goldberg 2006), a comprehensive understanding of plant functional connectivity in space is almost impossible to achieve. Measuring effective dispersal involves quantifying the establishment of new adults from dispersed seeds (Schupp et al. 2010), while evaluating the contribution of effective dispersal to upholding viable plant populations and communities in the face of habitat fragmentation would involve removing dispersal from a system entirely and measuring the subsequent biodiversity loss (Vandvik and Goldberg 2006). Instead, a number of indirect approaches have been used, which can give useful insights when considered together.

**Fig. 4:**
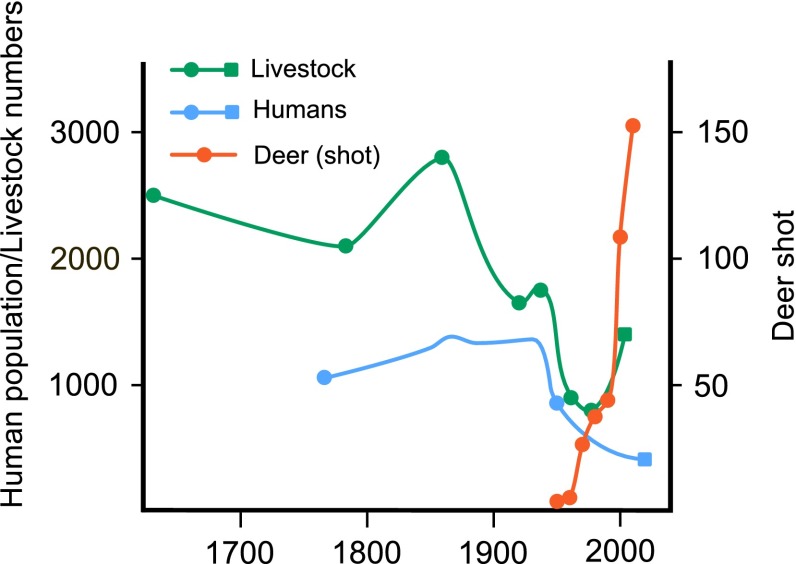
Populations of seed dispersal vectors across the parish of Överselö, Selaön (52 km^2^) based on available data between 1626 and 2014. Livestock (horses, cattle and sheep) are shown in absolute numbers (*left axis*) using data from Dahlström et al. (2006; 1626–1972—*circles*), apart from the most recent point taken from the 1999 Swedish agricultural register (*square*). Human populations (1760–1950) are redrawn from a figure without data points from Dahlström et al. (2006), circles indicate the beginning and end of this data series. The most recent point from 2014 was communicated by Strängnäs municipality (*square*). Deer data represent the number of animals (roe deer, fallow deer and red deer; *right axis*) registered shot at the finest available resolution for each year, adjusted to the area of Överselö (Auffret and Plue 2014)

Functional-trait approaches have indicated that the loss of structural connectivity has negatively affected functional connectivity, resulting in species loss and a reduction in traits relating to long-distance seed dispersal at the community level (Ozinga et al. 2009; Lindborg et al. 2012). On Selaön, Lindborg et al. (2014) found that the proportion of both animal-dispersed species and relative short-distance dispersers in remnant grassland communities decreased with increasing distance from the nearest intact semi-natural grassland. This is understandable, as increasing isolation from a species source should decrease the probability of successful dispersal, establishment, and potential replacement of species which may go locally extinct. This dispersal limitation is further supported by the fact that humans and livestock moving through the landscape have probably been valuable dispersers of grassland plant species in the past (Auffret 2011). On Selaön, both the human population and livestock numbers have generally declined alongside grassland loss (Fig. 4), and at the same time, their movement has become restricted through both management change and losses in structural connectivity.

However, a European meta-analysis recently found that species favored by dispersal by animals have been relatively resilient to losses in structural connectivity (Marini et al. 2012). Spatial functional connectivity through dispersal by humans and livestock has effectively disappeared on Selaön, but growing populations of roe deer (Fig. 4) have been found to disperse around a quarter of available plant species, including one-third of local grassland specialists (Auffret and Plue 2014). Furthermore, the small remnant habitats where deer are often found showed a relatively high proportion of animal-dispersed species in the established vegetation (Lindborg et al. 2014).

These apparently contradictory responses of functional connectivity to grassland fragmentation with regard to animal dispersal could be due to the extent to which the role of traditional human-mediated dispersal by livestock might be taken over by increasing numbers of wild animals (Fig. 4) or by rotational grazing for conservation management. Landscapes where there are large populations of wild ungulates or active rotational grazing networks might retain functional connectivity at the genetic and community level despite losses in structural connectivity (Rico et al. 2014; Auffret and Plue 2014). A further confounding factor is that while the numbers and movement of humans and livestock have declined in rural landscapes, a new type of disperser in the form of motor vehicles is able to move the same kinds of species and seeds as the traditional dispersers in the past (Auffret and Cousins 2013). However, the sheer number of livestock and people that moved across the historical landscape (Fig. 4) is probably difficult to replace with modern dispersal vectors. Furthermore, even if the presence of dispersal vectors means that the movement stage of seed dispersal is not necessarily always limiting in landscapes with low structural connectivity (Auffret et al. 2014), the subsequent stages of seed arrival and microsite limitation can still prevent effective functional connectivity (Schupp et al. 2010).

It is clear that considering the response of functional connectivity to losses in structural connectivity is vital. However, responses to land-use change occur not only in space, but also in time. Thus, the temporal element of functional connectivity is a further important aspect required for the understanding how plant communities respond to grassland loss and fragmentation.

#### Temporal functional connectivity

While the high species richness of semi-natural grasslands is related to the long-term, gradual accumulation of biodiversity through low-intensity management (Eriksson 2013), plant communities can also exhibit slow, gradual responses to negative environmental pressures such as land-use change (Jackson and Sax 2010). In semi-natural grassland communities, such temporal responses often follow the abandonment of grazing by livestock and resulting habitat fragmentation.

Grazing abandonment starts a gradual process of habitat degradation through the release of suppressed, competitive plant species, triggering the rapid competitive exclusion of disturbance-dependent species. This results first in a decline in habitat specialists, followed by more generalist grassland species as a forest canopy develops (Öckinger et al. 2006). This process of grazing abandonment occurred throughout Selaön during the 20th century (Figs. 1, 2). Nevertheless, plants have evolved a number of bet-hedging mechanisms which enable them to survive longer periods of unfavorable environmental conditions than might be expected following a loss of structural connectivity.

Two of these bet-hedging mechanisms, studied in the fragmented Selaön landscape (Plue and Cousins 2013; Lindborg et al. 2014), are crucial in sustaining temporal functional connectivity by enabling the long-term persistence of resistant life-cycle stages. First, persistence through perenniation and/or clonality is responsible for so-called remnant populations (Eriksson 1996), i.e., small populations of perennial plant species, which can temporarily escape extinction through clonal propagation (Honnay and Bossuyt 2005). Their presence in small, isolated, or abandoned grassland fragments can often be linked to historical grassland management. Moreover, besides harboring plant communities containing many grassland species (Lindborg et al. 2014), these small habitats and their remnant grassland populations can significantly increase community and ecosystem stability and resilience (Eriksson 1996; Cousins 2006). Over one-third (40 %) of grassland species in Selaön exhibit this bet-hedging mechanism (Plue and Cousins 2013). These are mainly grassland generalist species, whereas around 80 % of specialist and typical grassland species on fragmented and/or abandoned habitat fragments are able to disperse temporally through a second bet-hedging mechanism, namely storing persistent seeds in the seed bank (Plue and Cousins 2013). Although seed longevity has previously been linked to species occurrence patterns in fragmented systems (Ozinga et al. 2009), an empirical investigation of the seed banks of fragmented grassland patches on Selaön showed that seed banks indeed store numerous typical grassland species, often when the species are no longer present in the herb layer (Plue and Cousins 2013). Similar to remnant populations in the established vegetation, the presence of these banked species relates to the historical presence of semi-natural grassland and represents a potentially important demographic and genetic form of functional connectivity in time. The interaction of the two temporal functional connectivity mechanisms (clonality and seed banking) can potentially extend the lifespan of remnant populations and metapopulations, whereby persistent seeds can both strengthen remaining populations and rescue those which have gone locally extinct.

Although both mechanisms are able to maintain plant biodiversity, community and ecosystem stability, and resilience in fragmented landscapes, their prevalence means that grassland plant communities can be in disequilibrium with current landscape configuration (Lindborg and Eriksson 2004). Relying on clonal survival alone is an almost certain route to local extinction (Honnay and Bossuyt 2005), and seed banks deplete with time due to (failed) germination, seed predation, and seed senescence. Although temporal functional connectivity allows species to persist despite unsuitable conditions, these processes cannot maintain functional connectivity alone to prevent eventual local extinction.

## Discussion

Through studying and describing the spatial and temporal components of functional connectivity separately, it becomes clear that consideration of both is important in understanding the effects of changes in structural connectivity on plant communities today, as well as how present connectivity can be managed to meet the ongoing and future challenges of environmental change. Temporal functional connectivity of present-day plant communities has often resulted in diversity patterns being more strongly related to previous structural (and probably spatial functional) connectivity following habitat loss (e.g., Lindborg and Eriksson 2004; but see Cousins et al. 2015). This phenomenon provides an opportunity for conservation (Kuussaari et al. 2009), although time for action is finite. Therefore, it is important to improve grassland connectivity in space to avoid the looming threat of extinction.

The need for improving structural connectivity through habitat restoration is indisputable, but additionally considering both components of functional connectivity can contribute to making better-informed choices. Functional connectivity in space must be facilitated in order for target communities to (re)colonize. One method would be to create large pastures containing both pristine grassland and less species-rich modern grassland and abandoned remnant grassland habitats to both increase total habitat area and ensure a flow of seeds into target areas via free-ranging livestock (Kumm 2004). However, the risk exists that unfavorable generalists would disperse to the core habitat areas rather than vice versa (Mouissie et al. 2005). Alternatively, rotational grazing can provide more directed spatial functional connectivity for improved seed dispersal and gene flow between habitats (Auffret et al. 2012; Rico et al. 2014). More extreme measures for the restoration of modern grasslands such as topsoil removal and directed seed transfer can improve prospects for the colonization of target species (e.g., Rasran et al. 2007). However, while potentially improving the prospects for successful spatial functional connectivity, such measures would also eliminate any desirable species in the vegetation or seed bank which had already arrived at the site.

A consideration of the temporal aspect in restoration management also requires an understanding that time lags can exist in both directions, as time is also needed for species to (re)colonize restored or newly created habitats (Cristofoli et al. 2010; Jackson and Sax 2010). The extent of this delay will naturally depend on connectivity both in time and space. Temporal dispersers, i.e., species able to persist as clonal adults in more unfavorable conditions and those present in the soil seed bank should be able to establish quickly, whereas the extent of structural and spatial functional connectivity to the patch in question will determine how quickly colonization will occur.

For connectivity to be adequately integrated into conservation and restoration, it is necessary to consider the whole landscape in question, as landscape context is important in determining diversity and resilience in agricultural landscapes (Tscharntke et al. 2005). Small, remnant, and marginal habitats can be valuable in providing structural connectivity and facilitating functional connectivity in a landscape (Auffret and Cousins 2013; Auffret and Plue 2014). Furthermore, they can act as source habitats, accelerating diversification in restored areas (Cousins and Lindborg 2008; Auffret and Cousins 2011). Therefore, management of both core and marginal habitats should be appropriate to ensure that target species can set seed for subsequent dispersal and connectivity in both time and space (Auffret and Cousins 2011). Finally, connectivity can even be used to define the relevant spatial area for management of particular systems, ensuring that all aspects of connectivity are embedded in management actions to improve the likelihood of conservation success (Verhoeven et al. 2008).

## Conclusion

In this perspective, we have presented our case for considering both the temporal and spatial components of functional connectivity to understand and manage ecological communities in the face of changes in structural connectivity through land-use change and fragmentation. Specifically, we believe that the different aspects of temporal functional connectivity should receive more attention for their role in the ecological responses to change, and their feedbacks with the more established forms of connectivity related to spatial patterns. Although we focused on grassland habitats undergoing habitat destruction, temporal functional connectivity has been observed in a wide range of organisms in various habitats (Mergeay et al. 2007; Kuussaari et al. 2009), with species able to persist in a landscape despite losses in structural and spatial functional connectivity. Connectivity is also an issue for organisms responding to a warming climate, as structural and functional connectivity are required for species to track their climatic ranges to higher latitudes and altitudes (Hodgson et al. 2009). The temporal component of functional connectivity can allow species to persist despite a warmer, more variable climate (Plue et al. 2013; Hylander et al. 2015), or establish at new latitudinal and altitudinal limits following preemptive dispersal beyond current natural ranges (Molau and Larsson 2000; Van der Veken et al. 2008; Elmhagen et al. 2015).

Framing the temporal aspect of connectivity together with spatial aspects allows for a more holistic approach for understanding current patterns of diversity, predicting future responses to change, and planning conservation management. Although past geographic and biodiversity data might often be a limiting factor, recent advances in molecular methods could provide the potential for incorporating the temporal element into studies of connectivity. Finally, merely considering that connectivity can occur in both space and time and appreciating any relevant feedbacks and synergies would be a step in the right direction for a greater understanding and the effective management of organisms in a changing human environment.
